# Treatment of Traumatic Facial Atrophic Scars Using a Combined Laser Protocol Including Variable-Pulse Picosecond Technology: A Case Report

**DOI:** 10.7759/cureus.99987

**Published:** 2025-12-24

**Authors:** Martin L Castro, Pablo Russo, Paula Urcera, Alessandra Zevini, Daniela Martinelli, Riccardo Barini

**Affiliations:** 1 Dermatology, Clínica Integral Dr. Urcera, Neuquén, ARG; 2 Dermatology, Universidad Abierta Interamericana, Buenos Aires, ARG; 3 Clinical and Medical Affairs, El.En. Group, Calenzano, ITA

**Keywords:** atrophic scar, er:yag laser, facial trauma, nd:yag laser therapy, picosecond laser, scar remodeling, variable-pulse technology

## Abstract

The management of traumatic facial atrophic scars presents significant aesthetic and functional challenges. Traditional surgical approaches, while having documented efficacy, carry inherent risks of invasive procedures and the potential for creating secondary scarring. Therefore, this study aims to evaluate the effectiveness of a combined laser therapy approach, specifically utilizing potassium titanyl phosphate (KTP)/Nd:YAG, variable-pulse picosecond fractional laser, and Er:YAG lasers, as a non-surgical alternative for treating these complex traumatic scars.

A 42-year-old man presented with a 30-day-old atrophic facial scar resulting from a sharp-point trauma. Given the patient's desire to avoid surgical intervention, a multimodal laser protocol was initiated over four sequential sessions. The treatment targeted various aspects of the scar pathophysiology, including vascular remodeling (KTP), deep collagen stimulation (long-pulse Nd:YAG), fractional micro-injury (picosecond), and precise resurfacing (Er:YAG). A progressive and significant reduction in scar depth and thickness was achieved, as documented by a marked improvement in the Vancouver Scar Scale (VSS) score, which dropped from a baseline of 10 to a final score of 1. The procedure was characterized by minimal adverse effects and resulted in high patient satisfaction.

Combined laser therapy, integrating KTP/Nd:YAG, variable-pulse picosecond fractional, and Er:YAG lasers, represents a safe and effective alternative to surgical intervention for traumatic facial atrophic scars. This multimodal approach provides a means to achieve significant aesthetic improvement with minimal associated downtime and adverse effects. Further studies with larger patient populations and extended follow-up periods are warranted to confirm long-term efficacy and standardize treatment protocols.

## Introduction

Traumatic facial scars constitute a relevant clinical problem that significantly impacts patients' quality of life, leading to consequences that extend beyond simple aesthetic damage to include functional limitations and psychological repercussions. Beyond physical disfigurement, facial scarring is strongly associated with psychosocial distress, reduced self-esteem, social withdrawal, and even symptoms of anxiety and depression, all of which can profoundly impair daily functioning and interpersonal relationships [1].

Scar formation after penetrating trauma follows a precisely orchestrated cutaneous reparative process comprising three sequential biological stages [2]. The inflammatory phase, occurring in the first hours and days following the trauma, is characterized by vascular phenomena and the infiltration of immune cells, responsible for controlling hemorrhage and secreting soluble mediators that coordinate subsequent phases. This is followed by the proliferative phase, a period of several weeks dominated by neovascularization, fibroblast expansion, and the deposition of a new dermal matrix, parallel to the reconstitution of the epithelial covering. The process is completed over several months with the remodeling phase, characterized by the progressive maturation of the newly formed connective tissue, the reorganization of collagen fibers, and the regression of cellular and vascular components [3].

The timing of intervention is critical: early treatment during proliferative or early remodeling phases allows modulation of collagen architecture and vascular regression and offers a significantly higher probability of achieving favorable results [4]. Conversely, once the scar has reached complete maturation, the tissue architecture becomes increasingly resistant to modification, with therapeutic options becoming more limited and less effective.

Conservative measures, compression, silicone gels and sheeting, manual therapy, and injectable corticosteroids, remain the first-line treatments but show limited efficacy in stabilized or morphologically complex scars [5].

Surgical solutions, including excision, subcision, and autologous fat grafting, can achieve substantial improvements but carry the intrinsic risks of invasive procedures, require prolonged recovery times, and may result in variable outcomes based on individual healing capabilities [6].

Laser therapy has emerged as a cornerstone in the management of traumatic scars [7]. Ablative lasers, particularly CO₂ (10,600 nm) and Er:YAG (2940 nm), are historically considered the gold standard, with documented clinical improvement across a wide range of studies [8]. Their mechanism of action is based on selective photothermolysis, a principle first described by Anderson and Parrish, which refers to the precise destruction of a target chromophore through the absorption of light energy at a specific wavelength, while sparing surrounding tissue [9]. In the context of ablative lasers, the primary chromophore is water, which is abundant in both epidermal and dermal structures. When laser energy at wavelengths strongly absorbed by water (such as 10,600 nm for CO₂ and 2,940 nm for Er:YAG) is delivered in short pulses, it rapidly heats and vaporizes intracellular and extracellular water. This controlled thermal injury leads to ablation of scar tissue and induces a cascade of biological responses, including collagen denaturation, neocollagenesis, and dermal remodeling. Histological studies demonstrate significantly improved dermal collagen density, increased elastic fiber length, and enhanced skin texture following fractional Er:YAG and CO₂ laser treatments. The resulting architecture shows more organized collagen deposition and improved vascularization compared to untreated scar tissue [10].

Er:YAG offers advantages over CO₂, mainly attributable to reduced collateral thermal damage, which translates into shorter downtime, faster re-epithelialization, less postoperative pain, and milder persistent erythema [11]. However, critical issues remain, including the risk of edema, permanent pigmentary alterations (particularly in darker phototypes), and infectious complications, which limit their extensive use.

Non-ablative lasers provide a therapeutic compromise: while less effective than ablative lasers, they offer a superior tolerability profile; these tools are therefore elective for patients requiring a rapid return to daily activities or with skin characteristics predisposing to complications.

Optimal outcomes frequently derive from combining different laser modalities in concurrent or alternating treatment sessions. This integrated approach maximizes therapeutic benefits while minimizing side effects through complementary mechanisms.

We present a clinical case illustrating the efficacy of a combined protocol of ablative and non-ablative lasers for the treatment of a facial incisional trauma scar, demonstrating significant improvement in the functional, aesthetic, and symptomatic aspects.

## Case presentation

Patient information

A 42-year-old man with a Fitzpatrick IV skin type presented with a 30-day history of an untreated atrophic facial scar (104 mm in length) resulting from a sharp-point trauma. The patient reported no significant medical history and was not taking any medications that would contraindicate laser treatment.

Clinical examination revealed a well-demarcated atrophic scar with significant depth and irregular texture. The scar demonstrated characteristics typical of traumatic injury, including loss of tissue volume and altered skin architecture.

A structured questionnaire was administered to the patient to assess the psychosocial impact of the facial scar. The patient reported significant emotional distress, stating that the scar negatively affected daily life and created a persistent feeling of being observed by others. Self-perception had been markedly altered, with reduced self-confidence and discomfort in social settings. The patient acknowledged avoiding social events due to self-consciousness about the scar's visibility. While daily activities and work were generally not impaired, first-time social encounters were particularly challenging, as the patient perceived negative attention focused on the facial scarring.

Given the patient's desire to avoid surgical intervention and the scar's characteristics, a multimodal laser treatment approach was selected as the preferred therapeutic option.

Treatment protocol

The therapeutic approach employed a sequential multimodal laser treatment strategy, combining four different laser systems to address the various pathophysiological aspects of the atrophic scar.

The initial phase of each treatment session began with the potassium titanyl phosphate (KTP) laser (Ultralight, Quanta System S.p.A., Italy), utilizing fluence parameters ranging from 6 to 8 J/cm² with pulse durations between 10 and 16 milliseconds. A 5-mm spot size was employed with approximately 250 passes delivered at a 5-Hz repetition rate.

Following the KTP treatment, Nd:YAG laser therapy was administered with fluence settings between 10 and 16 J/cm² and shorter pulse durations of 1.2-1.5 milliseconds. Using the same 5-mm spot size, approximately 500 repetitions were delivered at a 10-Hz frequency.

The third component of the treatment protocol involved picosecond fractional laser technology (Discovery Pico Plus, Quanta System S.p.A., Italy) with an 8-mm spot size. Fluence parameters were carefully titrated between 0.2 and 0.55 J/cm², utilizing variable pulse durations ranging from 450 to 800 picoseconds. During each treatment session, the "long-medium-short" pulse duration modality was employed to target different dermal depths, ensuring comprehensive tissue remodeling throughout the dermal layers. Approximately 2,000 passes were performed to create controlled micro-injuries that would stimulate the natural wound healing cascade and promote new collagen formation.

The final phase of each treatment session employed Er:YAG laser (Twain 2940, Quanta System S.p.A., Italy) resurfacing with fluence settings between 1.5 and 2 J/cm² and a pulse duration of 0.3 milliseconds. A 9-mm microbeam spot size was utilized with a stacking technique, performing multiple passes (2-3 times) over the treatment area depending on the tissue response and healing progression observed.

The patient underwent a comprehensive treatment course consisting of four sessions, with six- to eight-week intervals between sessions to permit full tissue remodeling and collagen maturation. Throughout the treatment course, laser parameters were dynamically adjusted based on clinical response, tissue tolerance, and progressive improvement in scar characteristics.

Outcome and clinical observations

**Figure 1:**
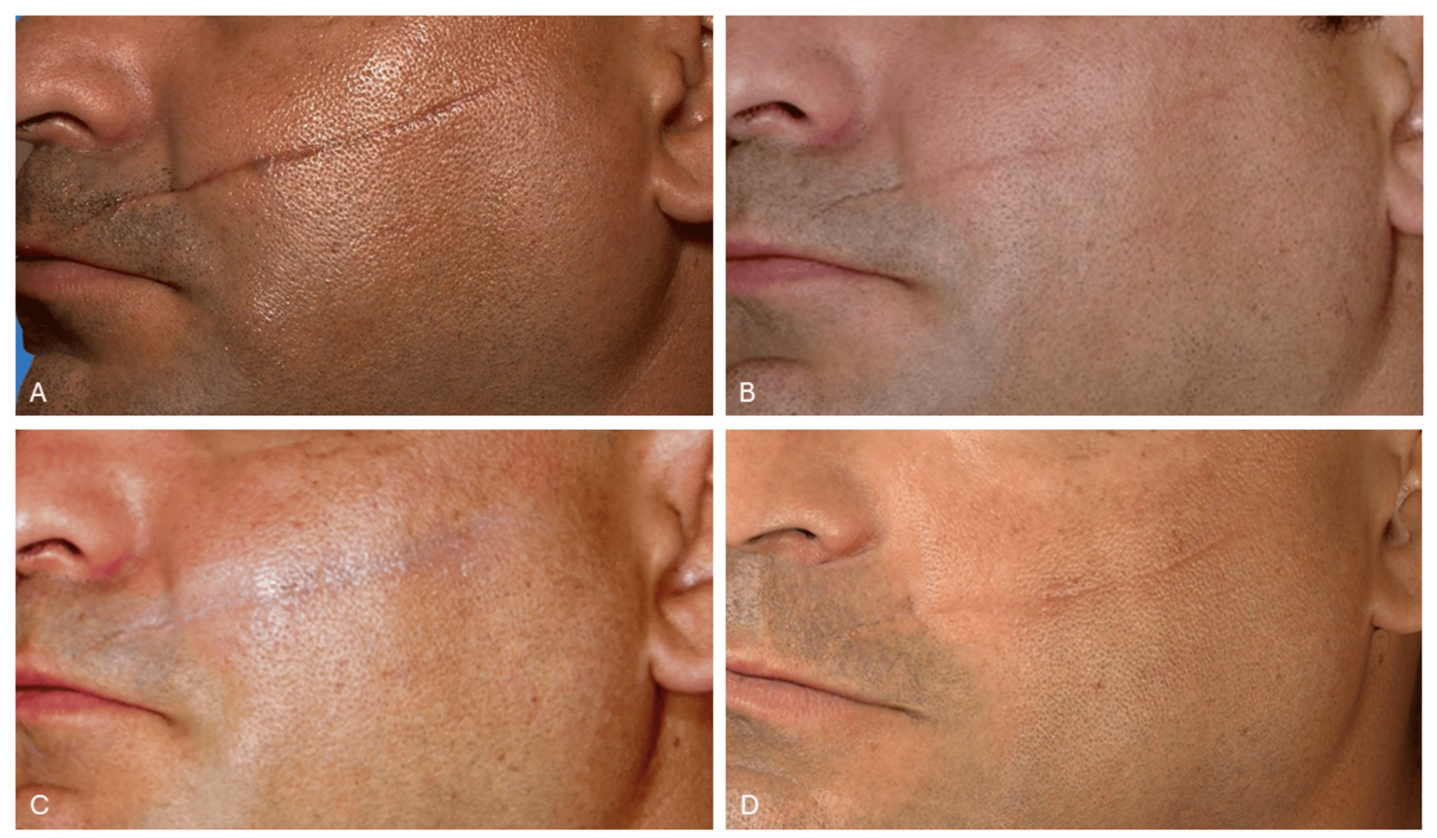
Clinical progression of traumatic facial atrophic scar treatment with combined laser therapy (A) Baseline presentation showing a 30-day-old atrophic scar. (B) Appearance after the second laser session. (C) Appearance after the third laser session. (D) Final follow-up at two months post-treatment showing substantial improvement.

Progressive and significant improvement was observed from the initial sessions, as documented by photographic follow‐up. After four treatment sessions, the results included a marked reduction in scar depth and improved skin texture and appearance (Figure 1).

The Vancouver Scar Scale (VSS) was utilized for objective clinical evaluation at baseline and post-treatment [12]. This scale, which assesses pigmentation (0-2), vascularity (0-3), pliability (0-5), and height (0-3), was used to quantify scar improvement. Since the VSS "height" parameter was originally designed for hypertrophic scars, it was adapted to reflect depth in this atrophic scar evaluation. The baseline VSS score was 11 out of a maximum of 13. After two laser sessions, a consistent reduction to a score of 7 was recorded, culminating in a score of 1/13 at the final follow-up (Table 1). Importantly, all individual parameters, pigmentation, vascularity, pliability, and depth, showed progressive and significant improvement throughout the treatment course, confirming the comprehensive efficacy of the multimodal laser approach.

**Table 1:** Vancouver Scar Scale (VSS) assessment at baseline, mid-treatment, and last follow-up

	Baseline	Mid-treatment	Follow-up
Pigmentation (0-2)	2	1	0
0 = Normal			
1 = Hypopigmentation			
2 = Hyperpigmentation			
Vascularity (0-3)	3	2	0
0 = Normal			
1 = Pink			
2 = Red			
3 = Purple			
Pliability (0-5)	4	3	1
0 = Normal			
1 = Supple			
2 = Yielding			
3 = Firm			
4 = Banding			
5 = Contracture			
Height (0-3)	2	1	0
0 = Normal (flat)			
1 = 0-2 mm			
2 = 2-5 mm			
3 = >5 mm			
Total	11	7	1

The patient was satisfied with the treatment result and concurred with the physician's assessment. He denied undergoing additional treatment beyond the four-session protocol.

**Figure 2:**
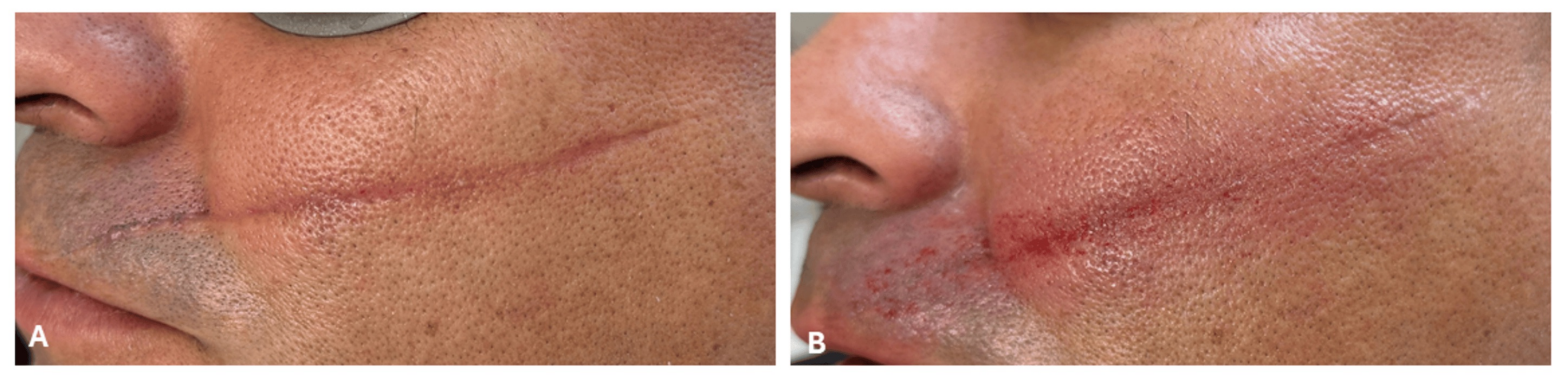
Immediate post-treatment appearance (A) Baseline presentation. (B) Immediate post-laser treatment response, showing expected acute inflammatory changes including erythema, edema, and mild purpura.

No long-term adverse events were observed. Figure 2 illustrates the immediate post-laser appearance. Following treatment, the patient exhibited expected acute inflammatory responses, including perilesional erythema, localized edema, and mild petechiae/purpura within the treatment zone. During the first 48 hours, transient pruritus was reported and successfully managed with emollient application. All acute side effects resolved within seven days without complications.

## Discussion

This case demonstrates the effectiveness of a combined laser therapy approach in treating traumatic facial atrophic scars. The multimodal strategy addresses various aspects of scar pathophysiology: vascular remodeling through KTP laser, collagen stimulation via long-pulsed and picosecond fractional Nd:YAG technology, and precise tissue ablation with Er:YAG laser resurfacing.

The initial use of the long-pulsed KTP laser is grounded in robust scientific evidence supporting its role in the vascular remodeling of scars [4,13-16]. Its 532-nm wavelength is particularly close to the absorption peak of oxyhemoglobin (542 nm), enabling selective photothermolysis of the scar's vascular components. Since vascular proliferation plays a key role in the early stages of scar formation, early intervention with the KTP laser can effectively interrupt this process. The use of long pulses (10-16 milliseconds) in our protocol was strategically chosen to induce controlled, gradual vascular damage, thereby limiting rapid vessel rupture and subsequent purpura formation.

The incorporation of a 1064-nm long-pulsed Nd:YAG laser as the second step in our sequential protocol addresses deeper structural components of atrophic scarring. Unlike the 532-nm wavelength, the 1064-nm laser penetrates significantly deeper into the dermis (5-10 mm), reaching the reticular dermis while relatively sparing the epidermis. This deeper penetration is crucial for targeting the fibrotic collagen architecture characteristic of scars [17]. Mechanistically, the long-pulse Nd:YAG laser induces non-ablative dermal wounding through selective photothermal damage, triggering expression of heat-shock protein 70 and upregulation of type I procollagen by dermal dendritic cells [18]. This thermal injury activates a cascade of wound healing responses, including the fibroblast-mediated release of inflammatory mediators and subsequent collagen remodeling. Prieto et al. demonstrated that Nd:YAG treatment results in increased collagen deposition in the papillary and upper reticular dermis, with significant improvements in fiber density and organization [18]. An important secondary benefit of the 1,064-nm laser is its vascular targeting capability, where broad oxyhemoglobin absorption helps suppress pathological neovascularization [19].

The third step involves the 1064-nm picosecond fractional Nd:YAG laser, bridging the gap between deep dermal remodeling (achieved with long-pulsed Nd:YAG) and superficial ablative resurfacing (achieved with Er:YAG laser). Picosecond pulses produce photomechanical and photoacoustic effects, creating laser-induced optical breakdown (LIOB) and controlled micro-injuries in the dermis without significant thermal diffusion to surrounding tissues [20]. Moreover, the fractional delivery pattern creates an array of microscopic treatment zones while preserving intervening areas of intact tissue, facilitating rapid healing through the reservoir of viable cells. This approach induces focal dermal necrosis, targeted disruption of abnormal collagen fibers, and subsequent neocollagenesis within the superficial to mid-dermal layers (1-3-mm depth), complementing the deeper remodeling achieved by long-pulsed Nd:YAG. In fact, the highly absorbing plasma generated during LIOB limits deeper beam penetration, concentrating the photomechanical effects primarily in the superficial to mid-dermal layers. This layered approach ensures comprehensive dermal restructuring: the long-pulse modality stimulates deep collagen deposition in the reticular dermis, while the fractional picosecond laser refines the papillary dermis and dermal-epidermal junction. The photomechanical disruption of rigid, cross-linked collagen bundles within the scar facilitates their replacement with more organized, elastic collagen architecture [21].

The vertical channels of thermal injury created by the fractional delivery pattern also enhance penetration and efficacy of the final Er:YAG laser resurfacing phase. This "priming" effect optimizes the overall regenerative response by pre-conditioning the scar tissue. The Er:YAG laser, with its 2940-nm wavelength and high water absorption, enables precise ablation with minimal thermal damage to surrounding tissues. The laser's mechanism of action involves two primary effects: vaporization of the epidermis, which stimulates natural healing through dermal appendages, and thermal stimulation of the dermis, promoting collagen remodeling and production [22].

The sequential use of different laser modalities created a synergistic effect, promoting natural healing responses while minimizing downtime and adverse effects. This laser approach provides notable advantages over surgical revision, primarily through its minimally invasive nature, which translates to a lower infection risk and reduced downtime. Furthermore, the superior precision in thermal modulation and tissue ablation lowers the risk of secondary scar formation [23].

The results obtained in this case report support the rationale for multimodal strategies already highlighted in the literature for surgical and hypertrophic scar management. Vas et al. demonstrated that the sequential combination of pulsed dye laser (PDL) (585 nm) and Nd:YAG is safe and effective in improving the appearance of surgical scars [24]. This efficacy was confirmed both clinically and structurally through in vivo confocal microscopy, showing reduced vascularization and reorganization of collagen fibers. The authors emphasized that combining two lasers with complementary mechanisms, selective vascular photothermolysis and dermal remodeling, yields superior results compared to single modalities, without significant adverse events [24].

Lee and Kim demonstrated excellent outcomes using sequential vascular laser (KTP 532 nm or PDL 585 nm) followed by 1550-nm fractional laser for immature post-surgical scars, achieving an average improvement score of 87.98 with 51.5% of cases showing complete resolution indistinguishable from surrounding normal skin [4].

Similarly, a recently published systematic review reported that both ablative fractional lasers (CO₂, Er:YAG) and PDL are effective for hypertrophic scars, but their combination reduces the number of sessions required and enhances overall efficacy [25].

Our multimodal approach demonstrated superior clinical outcomes compared to fractional Er:YAG monotherapy reported in the literature. While Osman et al. reported a median final VSS of 6 (range 2-8), with 40% of patients expressing neutral or negative satisfaction after four resurfacing sessions [26], our patient achieved a final VSS of 1, with high satisfaction after the same number of sessions. This difference may be attributed to the synergistic effect of combining vascular-targeting (KTP), deep dermal remodeling (Nd:YAG), pigment correction (picosecond), and ablative resurfacing (fractional Er:YAG) technologies, addressing multiple scar components simultaneously rather than texture alone. Notably, our combined protocol demonstrated a favorable safety profile. Compared to a graduated pulse duration fractional Er:YAG protocol, which reported elevated pain scores and significant adverse events, including 80% incidence of crusting and prolonged post-inflammatory hyperpigmentation (PIH) in some skin type IV patients [27], our treatment resulted only in transient edema, erythema, and purpura without crusting or PIH. This improved tolerability may reflect the lower fluences strategically employed in our protocol (1.5-2 J/cm²), substantially below both the study from Agamia et al. [27] and standard monotherapy settings. The multimodal synergistic approach enabled conservative energy settings for each individual wavelength while maintaining therapeutic efficacy through complementary mechanisms targeting distinct scar components. Moreover, the favorable safety profile observed in our Fitzpatrick IV patient may be partly attributed to the use of short pulses that confine thermal energy to the target chromophore and reduce heat diffusion to the surrounding melanin-rich epidermis, thereby lowering the risk of PIH in higher phototypes.

In our study, we treated a 30-day-old scar with a combination laser approach, recognizing that the onset of intervention is critical for optimal outcomes. The rationale for early treatment is grounded in wound healing physiology: initiating laser treatment during the inflammatory or early proliferation phase allows for theoretical modulation of the inflammatory cascade and alteration of fibroblast migration and activity (before the emergence of organized collagen fibers), which, together with improved microcirculation, may effectively prevent excessive scar formation and enhance final aesthetic appearance [28].

Several studies in the literature have emphasized the benefit of introducing laser treatments as early as possible. A 2018 systematic review found that the inflammation phase of wound healing represents the most beneficial time for laser initiation, as 3 out of 4 studies (75%) reported significant improvement when laser was applied during the inflammation phase, compared to 6 out of 16 studies (37.5%) in the proliferation phase and only 2 out of 5 studies (40%) in the remodeling phase [29]. 

Recent evidence suggests that the optimal window for scar treatment is within 2-4 weeks after injury [30], with some studies supporting the initiation of treatment within the first week, and no later than one month, post-operation to achieve the best results [31,32]. Our treatment timing falls within this recommended early intervention window, positioning our approach to maximally influence the ongoing remodeling process before scar maturation.

Despite the documented clinical improvement, this case report presents inherent methodological limitations. As a single-patient observation, the findings are limited in their generalizability and statistical power. Moreover, the lack of an untreated or monotherapy-treated comparator prevents the quantification of the individual contribution of each of the four laser modalities to the final result. Another limitation of this study is the relatively short follow-up of two months. However, the patient was monitored over a total period of nine months from the initial intervention, showing constant clinical improvement. The aforementioned literature supports our findings, indicating that multimodal laser protocols combining different wavelengths not only achieve superior outcomes but also synergistically accelerate the clinical response. By inducing early dermal remodeling, this approach provides a foundation for lasting stability. The consistent improvement documented throughout the nine-month study course supports the hypothesis that these results reflect genuine dermal restructuring rather than transient effects. Further studies with larger cohorts, control groups, and longer follow-up remain essential to establish standardized protocols and confirm the durability of the treatment efficacy.

## Conclusions

Combined laser therapy using KTP/Nd:YAG, picosecond fractional, and Er:YAG lasers represents a safe and effective treatment modality for traumatic facial atrophic scars. This multimodal approach achieved significant aesthetic improvement with minimal adverse effects and high patient satisfaction. Further studies with larger patient populations and extended follow-up periods are warranted to establish standardized treatment protocols and confirm long-term efficacy.
